# Call for papers for a special issue on how point of care/point of impact testing (POCT/ POIT) and self-testing impact surveillance and public health – deadline extended to 30 June 2019

**DOI:** 10.2807/1560-7917.ES.2019.24.22.1905301

**Published:** 2019-05-30

**Authors:** Eurosurveillance editorial team

**Affiliations:** 1 European Centre for Disease Prevention and Control (ECDC), Stockholm, Sweden

*Eurosurveillance* invites authors to submit papers in the categories Research, Surveillance, Outbreaks, Reviews and Perspectives for a special issue focusing on how point of care/point of impact testing (POCT/POIT) and self-testing impact surveillance and public health.

The submission deadline has been extended to **30 June 2019**.

Kits for testing close to the patient frequently by healthcare workers who may or may not have specific laboratory training, or self-testing are becoming increasingly available. Both POCT and POIT make testing easier and produce more rapid results, which enables timely treatment as well as the implementation of infection prevention and control measures. Self-testing approaches increase accessibility and confidentiality, which the individual may perceive as a benefit.

However, the incorrect use of tests can affect their accuracy and the quality, sensitivity and specificity of different tests can vary. Limitations of rapid diagnostics could have important consequences for individual and public health and thus appropriateness of use should be ensured. Aside from appropriate standards and accreditation of tests, self-tested patients need to be linked to care, and policies should be in place to address these varying aspects.

Capturing results from POCT/POIT and self-testing in a systematic and comprehensive manner may pose a challenge for traditional epidemiological surveillance. Moreover, for reference and public health laboratories, the possible limited availability of referred samples and/or isolates resulting from POCT/POIT and self-testing could impact on the detection of transmission pathways, source attribution and the detection of emerging new strains, antimicrobial resistance patterns etc.

This special issue aims to focus on the emerging role of POCT/ POIT and self-testing in contemporary communicable disease diagnostics, their utilisation and interpretation by public health experts and scientists and the range of benefits and challenges associated with their increased use.

Topics of interest may be, but are not limited to:

- The use of POCT/POIT and self-testing results in surveillance
- Illustration of solutions to improve linkage to care
- Possible role of POIT in outbreaks
- Possible role of POIT in populations moving across borders
- Possible role of rapid diagnostics in informing public health interventions (e.g. immunisation)
- Quality assurance, certification, accreditation in the public health context
- Policy and regulatory aspects of harnessing these new technologies for public health

If you would like to submit a paper or ask for more information, please see our instructions for authors regarding article formats or contact the editorial team at eurosurveillance@ecdc.europa.eu.

